# Iterative trocar-constrained laparoscope viewpoint search for enhanced surface coverage in 3D reconstruction

**DOI:** 10.1038/s41598-026-70217-x

**Published:** 2026-09-05

**Authors:** Birthe Wenking, Alexander Reiterer, Knut Möller

**Affiliations:** 1 Department of Sustainable Systems Engineering INATECH, University of Freiburg, 79110 Freiburg, Germany; 2 KARL STORZ SE & Co. KG, 78532 Tuttlingen, Germany; 3 Fraunhofer Institute for Physical Measurement Techniques IPM, 79110 Freiburg, Germany; 4 Institute of Technical Medicine (ITeM), Furtwangen University, 78054 Villingen-Schwenningen, Germany

**Keywords:** Laparoscope image-based 3D reconstruction, Stereo vision, Robot-assisted intervention, Hole repair, Computational biology and bioinformatics, Engineering, Mathematics and computing, Medical research

## Abstract

Autonomy is a growing trend in robot-assisted minimally invasive surgery. Autonomous systems require geometric information about the surgical environment, obtained from images and 3D reconstruction. The long-term goal of this work is to improve the completeness of endoscope-based 3D reconstruction by first creating a sparse 3D model and then guiding the laparoscope toward areas missing from the reconstruction. Guidance of rigid laparoscopes is challenging because the trocar, the entry point into the body, limits the degrees of freedom. Therefore, we propose an iterative geometric laparoscope viewpoint search approach, which considers the trocar point, the camera’s field of view, and the robot’s inverse kinematics. The validation, using ex vivo organ datasets containing prepared holes in a reconstructed point cloud, showed a success rate of 85%, in which viewpoints could be improved for the corresponding prepared holes. In contrast to existing methods applying interpolation to improve surface coverage, our method aims to cover feasible regions of interest with the camera. We achieved a reconstruction error of 1–2 mm with a relative improvement of the surface coverage of up to 6.3%. The computation time for the brute-force approach is 10 s on average per region of interest and could be reduced to 6 ms by applying a nonlinear optimization solver.

## Introduction

Robot-assisted surgery (RAS) has become an important part of modern operating rooms, with the da Vinci Surgical System (Intuitive Surgical, USA) being the most widely used system in minimally invasive laparoscopy. The surgeon operates via a console, offering advantages, such as improved ergonomics, compared to hand-held instruments. However, the da Vinci system does not perform any task autonomously, as pointed out by two review articles^1,2^. There, the authors classify surgical robots in five levels of autonomy, indicating a growing trend towards increasing autonomy. Tasks – such as autonomous endoscope movement – could be executed without direct surgeon control^3^ reducing the surgeon’s workload and intervention time. A possible application in laparoscopy could be a robotic camera assistant that guides the laparoscope entirely on its own instead of a surgeon^4^.

For a system to achieve autonomy, it must, among others, collect image data of the surgery scenery. Ref^5^. for example, emphasizes the importance of data-driven assistance in future robotic systems. Previous work, such as the STAR robotic system^6^, demonstrated the use of 3D reconstruction based on laparoscopic images for suture planning. Consequently, 3D reconstruction could act as an enabler for autonomous endoscope movement, which forms the motivation of this work: autonomous laparoscope navigation to visualize a certain region of interest (ROI) and to generate a complete 3D model of the operation scenery. To achieve complete reconstruction, view planning in active robot vision is the key^7^. Therefore, we developed a search-based approach to find candidate viewpoints based on the trocar point constraint. Then, the next-best view is selected from these candidates.

Next-Best-View (NBV) and viewpoint planning strategies for 3D reconstruction have been widely studied and some authors applied them on robot-assisted minimally invasive surgery (RMIS), as in Ref^8^. These methods are typically based on unconstrained camera motion and optimize viewpoints in full 6-DOF space. However, in RMIS, the laparoscope motion is restricted by the abdominal wall and the trocar point introducing the remote center of motion (RCM), which limits reachable viewpoints. As a result, directly applying existing NBV or viewpoint planning strategies to laparoscopic settings is not feasible.

## Constrained viewpoint search for hole repair in 3D reconstruction

**Fig. 1:**
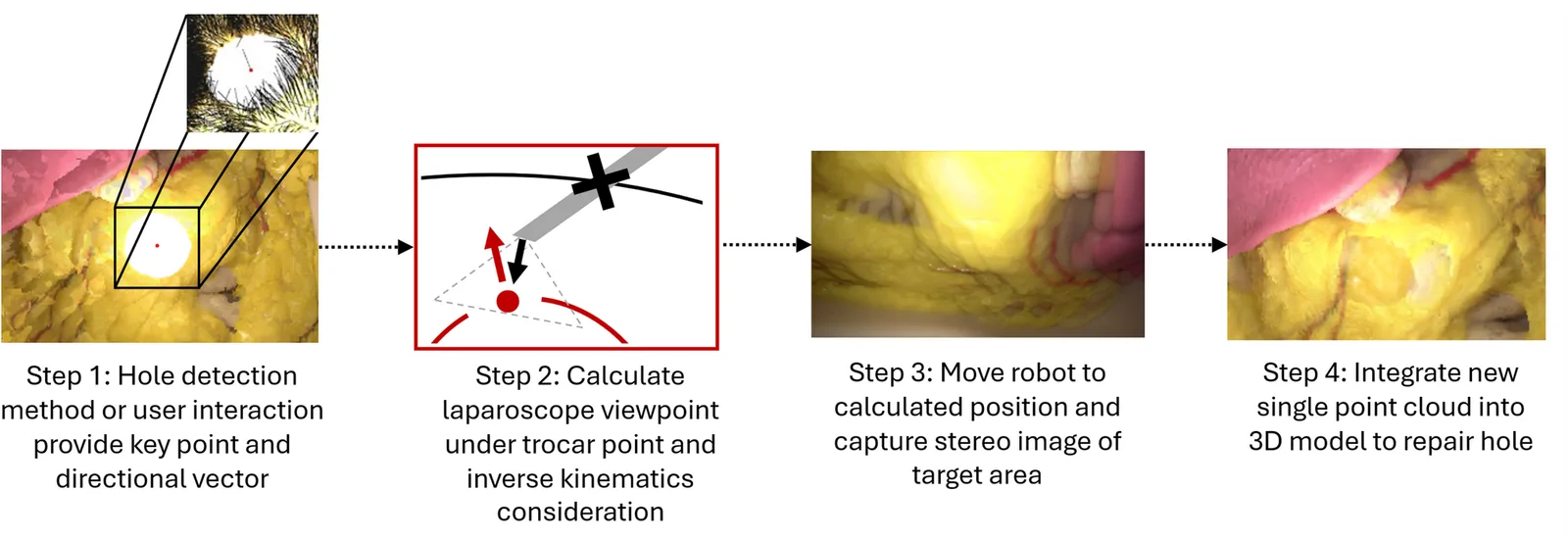
Overall process of hole repair including step 1, hole detection, step 2, iterative laparoscope viewpoint search, step 3, robot motion, and step 4, point cloud integration into 3D model for hole filling.

A rigid stereo laparoscope, which represents gold standard in laparoscopy, can provide the necessary data for accurate 3D reconstruction. However, its rigid shaft in combination with the trocar point introduces limitations and reduces the degrees of freedom from six to four, making autonomous endoscope movement challenging^9^ and leads to 3D reconstructions containing holes (Fig. 1, step 1, white circle represents a hole in a 3D model). Here, holes are not defined as actual physical holes in the object, but rather as areas in a 3D reconstruction that were not captured by the camera and are therefore missing. To tackle this challenge, our approach includes the calculation of laparoscope viewpoints that enable hole visualization and trocar point consideration based on a previously reconstructed 3D model.

This work builds upon two publications: one focused on generating 3D models from stereo images captured by a robot-guided laparoscope^10^, and the other on the automatic detection of holes in these models^11^(Fig. 1, step 1). The goal is to repair detected holes automatically and achieve a complete 3D model. Hole repair involves capturing new stereo images of the ROI (Fig. 1, step 3) and integrating those as individual point clouds into the incomplete model (Fig. 1, step 4). To accomplish this automatically, the robot must guide the laparoscope to the holes without manual intervention (Fig. 1, step 2). Following, we propose a hole repair pipeline that calculates laparoscope viewpoints, commands the robot to move, captures ROI images, and generates point clouds. The focus of this article is the iterative geometric laparoscope viewpoint search within this pipeline.

## Contributions

In this work, the authors present.

- a novel trocar-constrained laparoscope viewpoint search approach to enhance the surface coverage in a 3D reconstruction in robot-assisted laparoscopy formulated as a geometric problem. Rather than performing global exploration, the method specifically addresses holes in existing 3D reconstruction.
- experimental validation based on previously reconstructed ex vivo porcine organs resulting in point clouds containing prepared holes.
- a non-learning-based, purely geometric approach, which does not require training data.

The search solution outputs camera poses considering the robot’s inverse kinematics (IK), which are then executed to integrate new point clouds into the existing 3D reconstruction. The summarized research aim in Fig. 1 demonstrates an incomplete point cloud on the left and an enhanced point cloud after hole repair on the right.

## Related work

The primary goal of this work is to improve 3D reconstruction coverage with a high degree of autonomy. Therefore, the search for related work included repairing holes in 3D models, autonomous laparoscope navigation as well as viewpoint planning for 3D reconstruction. Existing approaches can be categorized into:

1. Data-driven hole filling methods.
2. Geometry-based interpolation methods.
3. Autonomous tool tracking and imitation learning.
4. Viewpoint planning and NBV strategies.

Data-driven hole filling methods plan on filling holes in 3D reconstructions using learned or statistical models. Reference^12^ presents a method for hole identification and segmentation in point clouds through density analysis and repairs holes based on a fuzzy system that incorporates geometry and feature information. Other examples of learning-based hole filling are based on a Point Fractal Network (PF-Net)^13^, or a combination of least squares generative adversarial network and spectral normalization (LSGAN and SN)^14^, or through identification of outlier points caused by reflections through a PointNet neural network (View-Transform-PointNet) and repairing of these areas through calculation of the distribution of outliers and structured light gray distribution^15^. Such learning-based methods can achieve plausible surface completions, but they require training data and the generalization to unknown surgical scenes, which remains challenging.

Geometry-based interpolation approaches try to complete 3D reconstruction by reusing existing data or by interpolating to fill missing gaps. Reference^16^. proposes a method for automatic hole detection and hole repair especially for symmetric objects. There, the authors distinguish between occlusions introduced by the grasper claw and missing surfaces resulting from limited imaging angles. Based on this classification, hole boundaries are set and partially interpolated. Holes caused by the grasper are repaired by surface reconstruction via Poisson reconstruction from the Point Cloud Library, whereas holes caused by limited viewpoints are repaired through symmetry completion (symmetric objects are considered exclusively). Similarly, Ref.^17^ focuses on interpolation-based hole filling, Ref.^18^ on identification of holes through hole boundaries and filling those by defining a polynomial as a best fit to the neighboring faces, and the authors of Ref.^19^ apply tensor voting to fill holes.

The methods share a common principle: they process and interpolate already available data to complete 3D reconstructions. While this performs well when no additional viewpoints can be acquired, one should first attempt completion through scene observation – especially in medical applications that require high levels of confidence to comply with regulations. In laparoscopy, the problem is not solely data completion, it is also a challenge to determine feasible viewpoints that allow the holes to be observed – and to do so autonomously.

In literature many different approaches are presented on how a laparoscope can be navigated autonomously by a robot. In contrast to our pipeline, many authors develop instrument tracking methods that lead to autonomous motion in laparoscopy, but which are dependent on the presence of instruments in the image. For example, in Refs.^20,21^ the laparoscope automatically follows the instrument motion under RCM-constraint. The technical approach of Ref.^20^ combines model predictive control (predicting future states of the laparoscope and instruments with the objective to keep the surgical instruments centered in the laparoscopic camera view) with dynamic waypoint generation (calculation of intermediate waypoints for smooth transitions). The technical approach of Ref.^21^ is based on the tracking of mark points on instrument arms and the authors also developed a forecasting kinematic algorithm to predict future positions with the Verhulst Grey Model for motion prediction. Both approaches show real-time control and smooth camera motion but are only applicable if the surgeon expects the instruments to be placed in the image center. Other examples of automatic instrument tracking can be found in Refs.^22–25^. Such methods are not applicable for hole repair in 3D reconstructions because it requires the detection of the holes and active guidance to those areas.

In other publications, the core idea is to leverage surgical expertise that is directly integrated into a learning pipeline, which is called imitation learning, as in Ref.^26^, where the authors present a framework that includes a robot performing gallbladder retraction under surgeon supervision. To do this, a robotic arm (UR5) holds a stereo laparoscope for 3D scene reconstruction, while a second robotic arm (Franka Emika Panda) moves the grasper for gallbladder resection. The initial step is for the surgeon to select landmarks, followed by a neural network-based prediction of the endpoints of the motion trajectory, and the generation of the motion trajectory based on Probabilistic Movement Primitives (ProMPs). Other examples within the categories imitation learning and learning from demonstration can be found in Refs.^4,27–30^. Such methods aim to enable the robot to take over resections tasks and replace the surgeon with task autonomy, but not with the goal of complete 3D reconstruction. Therefore, viewpoint planning and NBV strategies for 3D reconstruction could deliver promising results.

Classical Next-Best-View (NBV) methods want to maximize information gain or reduce incomplete reconstructions by selecting optimal viewpoints in unconstrained environments. These approaches include rule-based, sampling-based, and learning-based strategies, often assuming free camera placement and global scene exploration. In all the literature focusing on NBV planning for 3D reconstructions, applications like minimally invasive surgery (MIS) or laparoscopy can be rarely found. One example is the group of Su et al.^8,31^, who followed a larger ongoing research effort to reconstruct 3D surfaces using multiple viewpoints in robot-assisted minimally invasive surgery (RMIS). In Ref.^8^ they demonstrated and evaluated their approach of a context-aware autonomous camera positioning method with Ref.^31^ building up on their previous publication with a Deep Reinforcement Learning approach to garner improved viewpoint generation. In their work several cameras are fixed on the inner abdominal wall, which represents the constraint for candidate points. In contrast to our approach, the RCM constraint caused by the trocar point does not exist in their work.

In summary, existing work either focuses on reconstructing missing geometry from already existing data, selecting viewpoints under unconstrained conditions, or using alternative camera systems in laparoscopic scenes without RCM constraint. Our work explicitly aims to close this gap and therefore tackles the problem of viewpoint search for 3D reconstruction refinement under trocar-induced RCM constraints. Our approach combines geometric viewpoint search with constraints specific to laparoscopy.

## Methodology

**Fig. 2:**
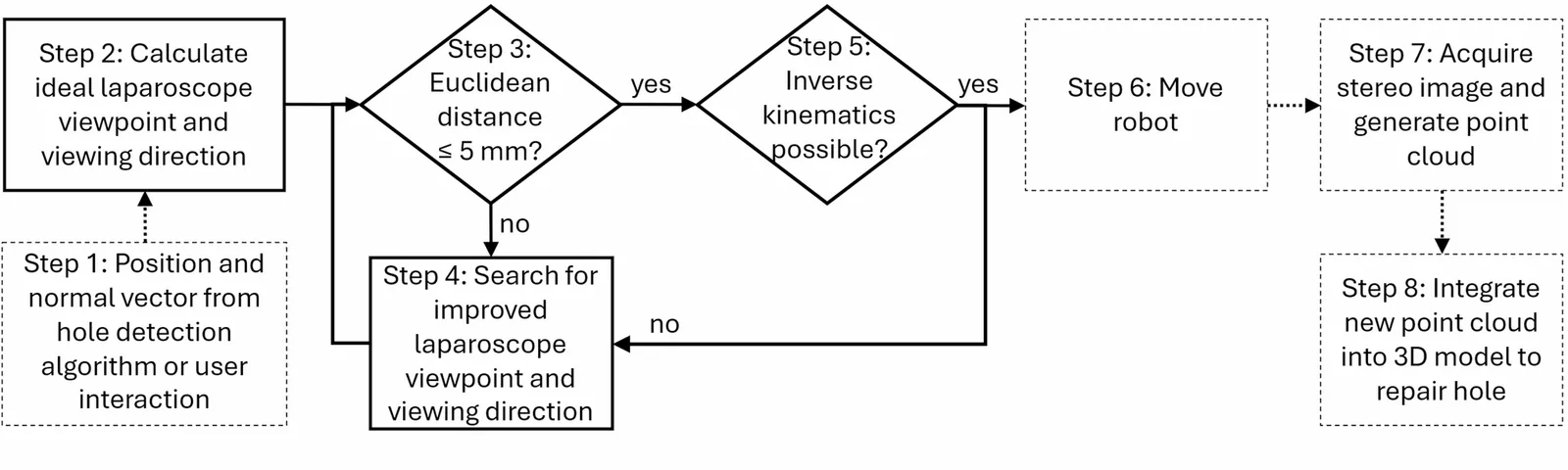
Laparoscope navigation pipeline including step 1–8, from defining the region of interest, calculation of ideal and improved laparoscope viewpoints, checking of inverse kinematics, robot motion, image acquisition, and point cloud integration. Steps 2–5 are focus of this work.

The approach generated for laparoscope navigation is illustrated in Fig. 2. The pipeline begins with a selected key point and corresponding normal vector as input (step 1). For each key point, the ideal laparoscope viewpoint is calculated (step 2). If the point is reachable under trocar point conditions and the key point lies in the image center with a positional error below five millimeters (step 3), the laparoscope viewpoint is selected. The five millimeters error tolerance results from the stereo camera’s overlapping FOV, which is a width of 63 mm and a height of 42 mm. The detected holes have a maximum diameter of 30 mm, which is covered by the stereo camera’s FOV even with a positional error of 5 mm. If the error exceeds the threshold, an improved laparoscope viewpoint is searched (step 4) such that the value falls below the threshold. As soon as a viewpoint is determined, the feasibility of inverse kinematics for the UR5 CB3 robot is evaluated (step 5) to verify if the robot can reach the position. If not, the search for an improved laparoscope viewpoint continues and the loop repeats. If yes, the command for movement (in joint space) can be sent (step 6), and a stereo image is captured from the selected viewpoint (step 7). The acquired stereo image leads to a point cloud, which can be integrated into the 3D model to repair holes (step 8).

All steps shown in dotted outlines (step 1, 6, 7, 8) are not core part of this work, for example step 8 is executed manually and will be automated in future contributions. The continuous, black-framed steps (step 2–5) represent the search algorithm and are the core part of this work. A detailed explanation of the sub steps is provided in the next sections. All algorithms necessary for this work – including robot motion commands, image acquisition, point cloud generation and stitching, hole detection, and viewpoint calculation – are implemented in Python including libraries such as OpenCV, Open3D, Numpy, rtde-ur.

Before explaining the search approach, we briefly introduce our system’s collision-risk-mitigation strategy between the laparoscope tip and organ tissue. The system checks candidate tip positions against the previously reconstructed mesh to reduce collision risk; however, this safety check depends on the accuracy and completeness of the initial reconstruction. Therefore, the point cloud from the initial scan is further processed into a watertight mesh by applying the Poisson surface reconstruction from the libraries Open3D and pymeshlab. Each laparoscope tip position is checked for collisions by measuring the distance between the tip and mesh, as well as detecting the occupancy (tip position inside or outside the mesh). For such use cases, the Open3D library provides the function called RaycastingScene.

**Fig. 3:**
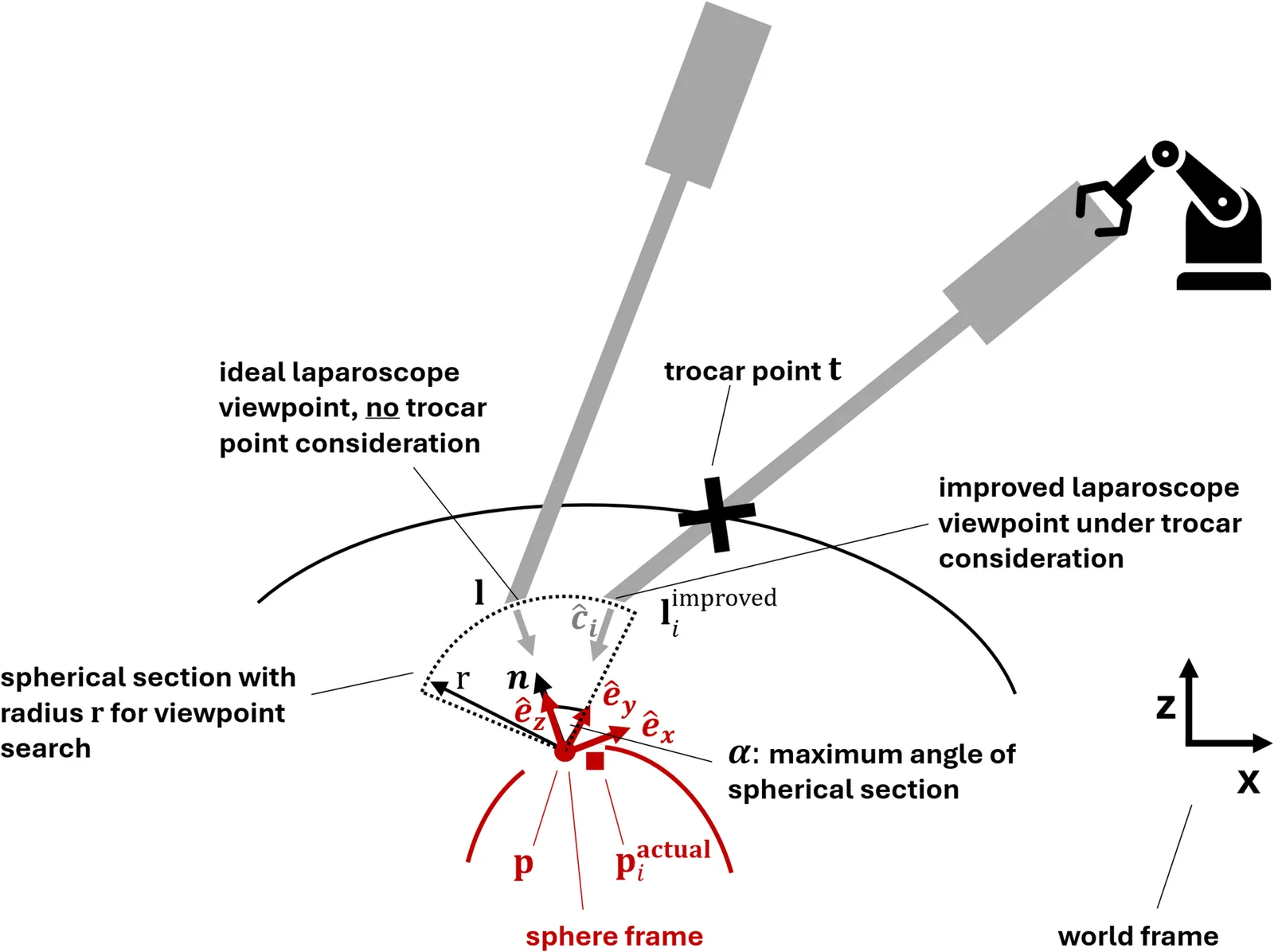
Sketch of spherical section-based laparoscope viewpoint search for capturing a region of interest defined by key point $\:\mathbf{p}$. The sketch shows the abdominal wall in black, the object with its key point $\:\mathbf{p}$ being the hole center, its directional vector $\:\boldsymbol{n}$, the sphere frame in red with its axes $\:{\widehat{e}}_{x},\:{\widehat{e}}_{y},{\widehat{e}}_{z}$, the world frame in black, the trocar point $\:\mathbf{t}$, and the laparoscope in grey with its ideal tip position $\:\mathbf{l}$, and its improved tip position $\:{\mathbf{l}}_{i}^{\mathrm{i}\mathrm{m}\mathrm{p}\mathrm{r}\mathrm{o}\mathrm{v}\mathrm{e}\mathrm{d}}$. It demonstrates the improved laparoscope viewpoint $\:{\widehat{\boldsymbol{c}}}_{\boldsymbol{i}}$, visualizing the new key point $\:{\mathbf{p}}_{i}^{\mathrm{a}\mathrm{c}\mathrm{t}\mathrm{u}\mathrm{a}\mathrm{l}}$, which can deviate from the original key point $\:\mathbf{p}$ by up to 5 mm. The maximum for the angle $\:\alpha\:$ is 45° and the sphere radius $\:\mathrm{r}$ is 50 mm.

In this work, a ROI is defined by a key point and its normal vector within a 3D model. The normal vector is orthogonal to the ROI’s surface and acts as a direction vector. Ideally, the laparoscope is positioned in a way that the key point lies in the center of the camera’s FoV, while the camera’s viewing direction points towards the opposite of the normal vector. Figure 3 illustrates the geometric relations between the laparoscope (grey), its viewing direction (grey arrow), the trocar point $\:\mathbf{t}$, the key point $\:\mathbf{p}$, and normal vector $\:\boldsymbol{n}$. All coordinates are defined with respect to the robot’s world frame (black frame). A local frame called sphere frame is introduced with its origin at the key point position and its z-axis aligned with the normal vector (red frame). The ideal laparoscope viewpoint is calculated according to Eq. (1) with radius $\:\mathrm{r}=50\:\mathrm{m}\mathrm{m}$ and the unit z-axis direction $\:{\widehat{\boldsymbol{e}}}_{\boldsymbol{z}}$. A radius of 50 mm provides the optimal distance between the laparoscope tip and the anatomical structure due to the intrinsic camera parameters. The threshold of 50 mm results from the compromise between high-resolution imaging, while ensuring enough safety distance to avoid collisions.

1 $${\mathbf{l}}={\mathbf{p}}+{\mathbf{r}}{\hat{\boldsymbol{e}}}_{\boldsymbol{z}}$$

Figure 3 presents a scenario, in which the ideal laparoscope viewpoint (Fig. 3, left laparoscope) must be improved resulting in a new laparoscope viewpoint (right laparoscope). The search approach is based on a spherical section, whose surface defines the search space for potential laparoscope viewpoints. In each iteration, all surface points are evaluated to determine whether both the trocar point and the key point image centricity are satisfied through one laparoscope viewpoint. The laparoscope viewpoint $\:{\mathbf{l}}_{i}^{\mathrm{i}\mathrm{m}\mathrm{p}\mathrm{r}\mathrm{o}\mathrm{v}\mathrm{e}\mathrm{d}}$ must be improved with the objective of minimizing the Euclidean distance between the key point $\:\mathbf{p}$ and the actual image center point $\:{\mathbf{p}}_{i}^{\mathrm{a}\mathrm{c}\mathrm{t}\mathrm{u}\mathrm{a}\mathrm{l}}$ seen from $\:{\mathbf{l}}_{i}^{\mathrm{i}\mathrm{m}\mathrm{p}\mathrm{r}\mathrm{o}\mathrm{v}\mathrm{e}\mathrm{d}}$.

The improved laparoscope viewpoint $\:{\mathbf{l}}_{i}^{\mathrm{i}\mathrm{m}\mathrm{p}\mathrm{r}\mathrm{o}\mathrm{v}\mathrm{e}\mathrm{d}}$ is calculated by iterating * i* times with step size 1° over all points on the spherical section’s surface by applying Eq. (2), with key point $\:\mathbf{p}$, radius $\:\mathrm{r}=50\:\mathrm{m}\mathrm{m}$, rotation matrix $\:{{}^{\mathrm{w}\mathrm{o}\mathrm{r}\mathrm{l}\mathrm{d}}\mathbf{R}}_{\mathrm{s}\mathrm{p}\mathrm{h}\mathrm{e}\mathrm{r}\mathrm{e}}^{}$ (rotation from world frame into the sphere frame), and the potential points on the spherical section surface $\:{\mathbf{s}}_{i}$. The derivation of the rotation matrix $\:{{}^{\mathrm{w}\mathrm{o}\mathrm{r}\mathrm{l}\mathrm{d}}\mathbf{R}}_{\mathrm{s}\mathrm{p}\mathrm{h}\mathrm{e}\mathrm{r}\mathrm{e}}^{}$ is demonstrated in the Appendix.

2 $$\:{\mathbf{l}}_{i}^{\mathrm{i}\mathrm{m}\mathrm{p}\mathrm{r}\mathrm{o}\mathrm{v}\mathrm{e}\mathrm{d}}=\mathbf{p}+{{}^{\mathrm{w}\mathrm{o}\mathrm{r}\mathrm{l}\mathrm{d}}\mathbf{R}}_{\mathrm{s}\mathrm{p}\mathrm{h}\mathrm{e}\mathrm{r}\mathrm{e}}^{}\:{\mathbf{s}}_{i}$$

The spherical section surface $\:{\mathbf{s}}_{i}$ lies within the sphere frame $\:{\mathcal{F}}^{\mathrm{s}\mathrm{p}\mathrm{h}\mathrm{e}\mathrm{r}\mathrm{e}}$, as presented in Eq. (3). The function $\:{f}_{\mathrm{s}\mathrm{p}\mathrm{h}\mathrm{e}\mathrm{r}\mathrm{e}}\left(\mathrm{r},\:{\theta\:}_{i},{\phi\:}_{i}\right)$ contains the radius $\:\mathrm{r}$ and the angular bounds $\:{\theta\:}_{i},{\phi\:}_{i}$.

3 $$\:{\mathbf{s}}_{i}={f}_{\mathrm{s}\mathrm{p}\mathrm{h}\mathrm{e}\mathrm{r}\mathrm{e}}\left(\mathrm{r},\:{\theta\:}_{i},{\phi\:}_{i}\right)\in\:{\mathcal{F}}^{\mathrm{s}\mathrm{p}\mathrm{h}\mathrm{e}\mathrm{r}\mathrm{e}}$$

The spherical section is limited to 45°, meaning $\:\theta\:\in\:[0^\circ\:,\:45^\circ\:],$ to ensure sufficient ROI visibility (a viewing direction from higher angles might not visualize the ROI well enough or the ROI is occluded by neighboring anatomical structure), while $\:\phi\:\in\:[0^\circ\:,\:360^\circ\:]$, both $\:\theta\:$ and $\:\phi\:$ with a step size of 1°. We choose a brute-force search approach to guarantee that a solution for each ROI can be found regardless of its computational efficiency.

For each $\:{\mathbf{l}}_{i}^{\mathrm{i}\mathrm{m}\mathrm{p}\mathrm{r}\mathrm{o}\mathrm{v}\mathrm{e}\mathrm{d}}$, the corresponding laparoscope unit axis direction $\:{\widehat{\boldsymbol{l}}}_{i}$ is determined through Eq. (4), calculating the vector from trocar point $\:\mathbf{t}$ to $\:{\mathbf{l}}_{i}^{\mathrm{i}\mathrm{m}\mathrm{p}\mathrm{r}\mathrm{o}\mathrm{v}\mathrm{e}\mathrm{d}}$.

4 $$\:{\widehat{\boldsymbol{l}}}_{i}=\left|{\mathbf{l}}_{i}^{\mathrm{i}\mathrm{m}\mathrm{p}\mathrm{r}\mathrm{o}\mathrm{v}\mathrm{e}\mathrm{d}}-\mathbf{t}\right|$$

From there, the camera viewing direction is defined through the unit vector $\:{\widehat{\mathbf{c}}}_{\boldsymbol{i}}$, which is $\:{\widehat{\boldsymbol{l}}}_{i}$ rotated by the rotation matrix $\:{{}^{\mathrm{w}\mathrm{o}\mathrm{r}\mathrm{l}\mathrm{d}}\mathbf{R}}_{\mathrm{l}\rightarrow\mathrm{c}\mathrm{a}\mathrm{m}}^{}$ (Eq. 5). The rotation is composed of two rotations: Rotation from flange to camera – known from the hand-eye-calibration – and rotation around the endoscope axis by angle $\:\psi\:$, which is improved iteratively with step size 1°. The hand-eye-calibration is based on the fixed mechanical mounting of the laparoscope onto the robot flange.

5 $$\:{\widehat{\boldsymbol{c}}}_{i}=\:{{}^{\mathrm{w}\mathrm{o}\mathrm{r}\mathrm{l}\mathrm{d}}\mathbf{R}}_{\mathrm{l}\rightarrow\mathrm{c}\mathrm{a}\mathrm{m}}^{}\:{\widehat{\boldsymbol{l}}}_{i}$$

Finally, the point $\:{\mathbf{p}}_{i}^{\mathrm{a}\mathrm{c}\mathrm{t}\mathrm{u}\mathrm{a}\mathrm{l}}$ can be determined (Eq. 6), which represents the actual image center point seen from $\:{\mathbf{l}}_{i}^{\mathrm{o}\mathrm{p}\mathrm{t}}$.

6 $$\:{\mathbf{p}}_{i}^{\mathrm{a}\mathrm{c}\mathrm{t}\mathrm{u}\mathrm{a}\mathrm{l}}=\:{\mathbf{l}}_{i}^{\mathrm{i}\mathrm{m}\mathrm{p}\mathrm{r}\mathrm{o}\mathrm{v}\mathrm{e}\mathrm{d}}+\:\mathrm{r}\:{\widehat{\boldsymbol{c}}}_{\mathrm{i}}$$

The iteration ends as soon as a first valid solution is found, meaning the objective that the Euclidean distance between the point $\:{\mathbf{p}}_{i}^{\mathrm{a}\mathrm{c}\mathrm{t}\mathrm{u}\mathrm{a}\mathrm{l}}$ and the original point $\:\mathbf{p}$ is below 5 mm is achieved, as in Eq. (7).

7 $$\:{||{\mathbf{p}}_{i}^{\mathrm{a}\mathrm{c}\mathrm{t}\mathrm{u}\mathrm{a}\mathrm{l}}-\mathbf{p}||}_{2}<5\:mm$$

The calculation of the UR5’s inverse kinematics is done by applying the inverse kinematic solver based on Ref.^32^.

The pipeline is summarized with the following pseudocode:

**Algorithm 1:** Laparoscope tip position search and robot pose generation.

Input: hole center position p, normal vector n, trocar position t, radius r
Output: Final tip position L_final, robot motion pose robot_pose
1: L ← calculate_L_ideal(p, r, n) // referring to Eq. (1)
2: p_actual ← project_p_actual(L, t, p, r)
2: IF d(p_actual, p) < threshold THEN
3: L_final ← L
4: ELSE
5: Initialize angular search with step sizes Δθ = 1° and Δϕ = 1°
6: REPEAT
7: s_i = f_sphere(r, θ_i, ϕ_i) // referring to Eq. (3)
8: L_i^improved ← calculate_L_improved(p, R_sphere, s_i) // referring to Eq. (2)
9: p_i^actual ← Project_p_actual(L_i^improved, t, R_cam, r) // referring to Eqs. (4–6)
10: IF d(p_i^actual, p) < threshold THEN // referring to Eq. (7)
11: L_final ← L_improved
12: EXIT loop
13: END IF
14: Update θ_i and ϕ_i by 1°
15: UNTIL θ_i = 45° and ϕ_i = [0°, 360°]
16: END IF
17: robot_pose ← rob_pose(L_final, t, p)
18: IF inverse_kin(robot_pose) = = TRUE THEN
19: RETURN L_final, robot_pose
20: ELSE
21: Restart angular search
22: END IF

For validation, three quantitative metrics are applied, and the resulting point clouds are evaluated qualitatively regarding their appearance. The first metric is the number of improved viewpoints relative to the number of holes in percent, the second metric is the surface coverage in percent, and the third the reconstruction error expressed as root-mean squared error (RMSE) in millimeters. The reconstruction error is calculated as in Eq. (8) with $\:{d}_{i}$ being the distance between reconstructed points and corresponding closest points on the ground truth surface, and the number of scanned points * N*.

8 $$\:\mathrm{R}\mathrm{M}\mathrm{S}\mathrm{E}=\sqrt{\frac{1}{N}\sum\limits_{i=1}^{N}{d}_{i}^{2}}$$

The surface coverage indicates how much of the real surface is covered by reconstruction and it is expressed by Eq. (9) with * g* as the points on the ground truth, * recon* as the corresponding points on the reconstructed surface, all ground truth points * GT* and the threshold $\:\tau\:$. If the distance between ground truth and reconstructed point is below the threshold of 2 mm, that point is counted. The threshold is chosen to be within the range of the RMSE of the reconstruction error, which lies in our cases typically between 1 and 2 mm.

9 $$\:\mathrm{c}\mathrm{o}\mathrm{v}\mathrm{e}\mathrm{r}\mathrm{a}\mathrm{g}\mathrm{e}=\:\frac{\left|\left\{g\in\:GT\right.\right.:\:d(g,\:recon)<\left.\left.\tau\:\right\}\right|}{\left|GT\right|}\:\times\:100$$

Before both RMSE and coverage can be computed, the reconstructed point cloud must be registered to the ground truth point cloud, which we do manually in the open-source software program CloudCompare. Two steps are executed in CloudCompare: First, four point pairs are picked manually in the point clouds for a rough alignment and secondly, fine registration through iterative closest point (ICP) is done. The registration logic for integrating new scans for hole repair is based on the robot’s position, which is recorded during the scan process, and which is taken for rough alignment, followed by a fine registration through ICP in CloudCompare and optionally combined with a manual adjustment.

## Experimental set-up

**Fig. 4:**
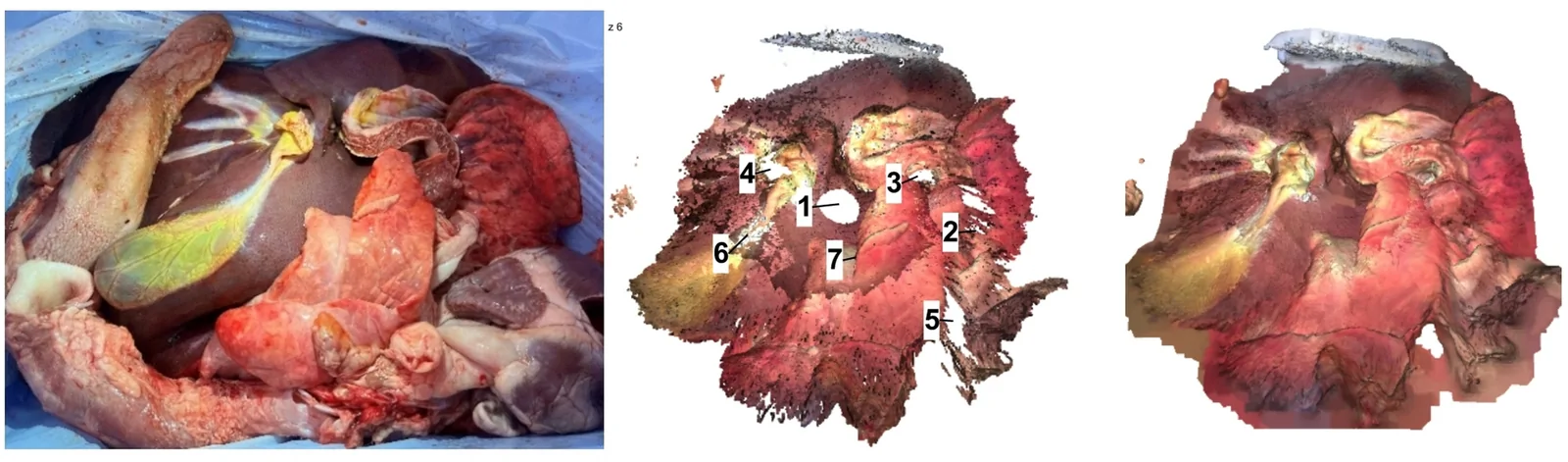
Photograph of porcine ex vivo organs used for preparation of the datasets (left), the point cloud after applying a simple scanning trajectory containing seven prepared holes (middle), the corresponding mesh without hole repair generated through Poisson surface reconstruction, hole filling is only done by surface reconstruction (right).

This section introduces the equipment used for this work, followed by the section, which presents the results of iterative laparoscope viewpoint search. The experimental set-up includes the TipCam Rubina 1 S 30° stereo laparoscope (KARL STORZ SE & Co. KG, Tuttlingen, Germany), a UR5 CB3 robotic arm (Universal Robots A/S, Odense, Denmark) for laparoscope guidance, a Dell Tower Precision 5820 with two NVIDIA RTX A4000 graphic cards for computation. Image acquisition was performed using the Image 1 S camera control unit (KARL STORZ SE & Co. KG, Tuttlingen, Germany) in combination with the USB 4 K framegrabber (Nanjing Magewell Electronics Co.,Ltd, Nanjing, China). Ex vivo porcine organs (heart, liver, gallbladder, lungs, tongue, and trachea) sourced as by-products from a meat market (Metzgerei Becker GmbH, Tuttlingen, Germany) were used for validation experiments (Fig. 4, left). Rough 3D reconstructions of these organs were previously generated containing holes that need to be repaired (Fig. 4, screenshot of point cloud in the middle, white areas, representing holes, are numbered). Without hole repair, the poisson surface reconstruction from Open3D library interpolates these areas (Fig. 4, screenshot of mesh on the right), which might lead to wrong assumptions. We distinguish between a point cloud and a mesh. A mesh is a closed surface model (often triangle surfaces connecting points), which is generated based on a point cloud through surface reconstruction. Our approach works with point clouds.

The approach was evaluated on fourteen different point clouds with each having an average resolution of 0.12 mm and a density of 69 pts/mm^2^, which required preparatory work containing the following steps: build experimental set-up (place robot, laparoscope and organs), define a robot trajectory for rough 3D reconstruction, follow robot trajectory for image acquisition, stitch individual point clouds to combine them to one whole, detect holes in the 3D reconstruction.

First, a virtual trocar was positioned around 15 centimeters above the box containing the organs, representing the typical distance between organs and abdominal wall for an average patient. The trocar was also positioned centrally above the organ box, but this position was varied by a few centimeters to generate different point cloud scenarios. The trocar point is calibrated by positioning the laparoscope tip in the desired location and then recording the current location of the robot flange. From there, we know the position of the laparoscope tip through the hand-eye calibration described in the section Methodology. The robot trajectory for 3D reconstruction consisted of two circular paths, each with 25 images. The point clouds were stitched as detailed in Ref.^10^, while the hole detection was performed according to Ref.^11^. Finally, the basis for the validation of our iterative viewpoint search is available.

## Results

Table 1 summarizes the results of the fourteen evaluated point clouds. The first column lists the point cloud number. All point clouds were reconstructed from ex vivo porcine organ images except for point cloud #1, which was reconstructed from images of laparoscopic dummy organs. The second column states the number of prepared holes, which were detected as part of the preparatory work. The third column states the number of successfully improved laparoscope viewpoints, which cannot exceed the number of prepared holes (one improved viewpoint per hole). The fourth column gives the number of improved viewpoints that could not be reached by the UR5 CB3 robot, which we call unreachable viewpoints due to unsuccessful inverse kinematics (IK) results.

**Table 1:** Summary of the evaluation results for 14 different point clouds of ex vivo porcine organs, listing the number of prepared holes, number of successfully improved viewpoints, the number of unreachable viewpoints due to unsolvable inverse kinematics.

Point cloud	Prepared holes	Improved viewpoints	Unreachable viewpoints (no IK)
#1	4	3	0
#2	7	5	0
#3	6	5	0
#4	5	4	0
#5	7	6	0
#6	9	9	0
#7	8	7	0
#8	2	1	0
#9	3	3	0
#10	10	7	0
#11	5	5	0
#12	4	4	0
#13	1	1	0
#14	4	4	0

**Fig. 5:**
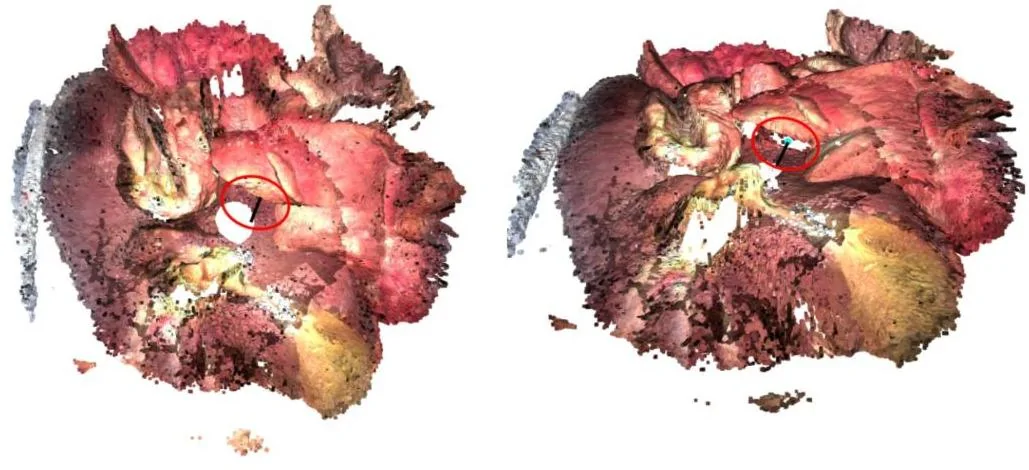
Screenshot of point cloud #5 from the front view (left) and a side view (right) to visualize hole number 7 with the hole center (turquoise) and its corresponding normal vector (black). The hole is outlined in red.

In case of point cloud #5, our approach finds an improved solution for six of seven prepared holes (holes number 1–6 displayed in Fig. 4), while it could not find a solution for hole number 7 (displayed in Figs. 4 and 5). For all six improved viewpoints, the IK calculator could find a solution for the UR5’s inverse kinematics. Overall, most (85%) laparoscope viewpoints could be successfully improved in all fourteen datasets, and none of them (0%) are unreachable due to a lack of inverse kinematic solutions.

**Fig. 6:**
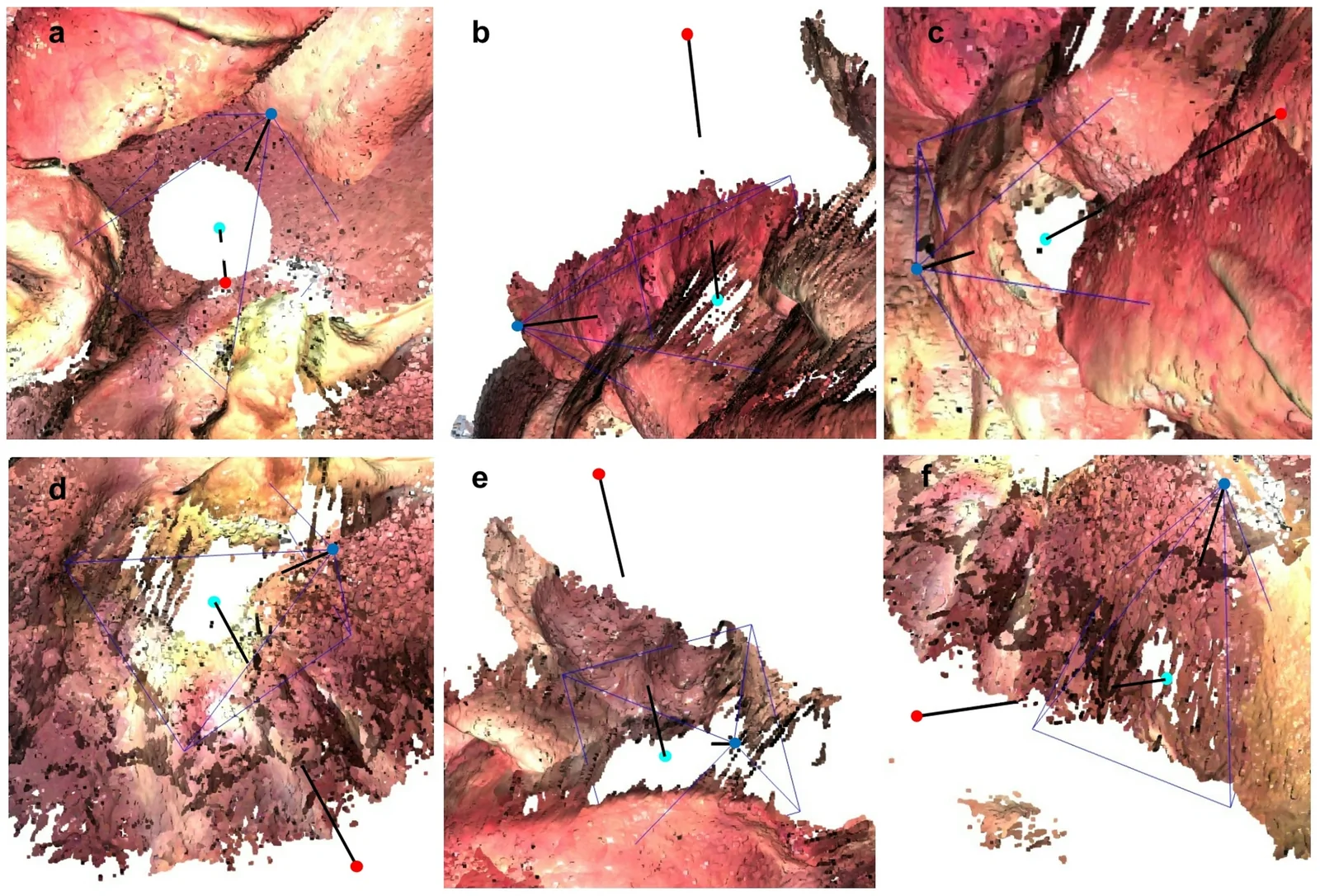
Screenshot of point cloud #5 with a partial front view at hole number 1 (**a**), at hole number 2 (**b**), at hole number 3 (**c**), at hole number 4 (**d**), at hole number 5 (**e**), at hole number 6 (**f**). In all six partial views the following information is marked in colors: the detected hole centers (turquoise), corresponding normal vectors (black), ideal laparoscope viewpoint (red) and viewing direction (black), and the improved laparoscope viewpoint including FOV pyramid (blue) and viewing direction (black).

Figure 6 presents partial views of the point cloud #5 to focus on each hole separately. The partial views emphasize the improved laparoscope viewpoints (blue), their corresponding FoV (blue pyramid), the ideal laparoscope viewpoints (red), the hole centers (turquoise), and all direction vectors (black). The results are visualized by applying the visualization library from Open3d. For better visibility, we increased the markers representing points and vectors by using PowerPoint.

**Fig. 7:**
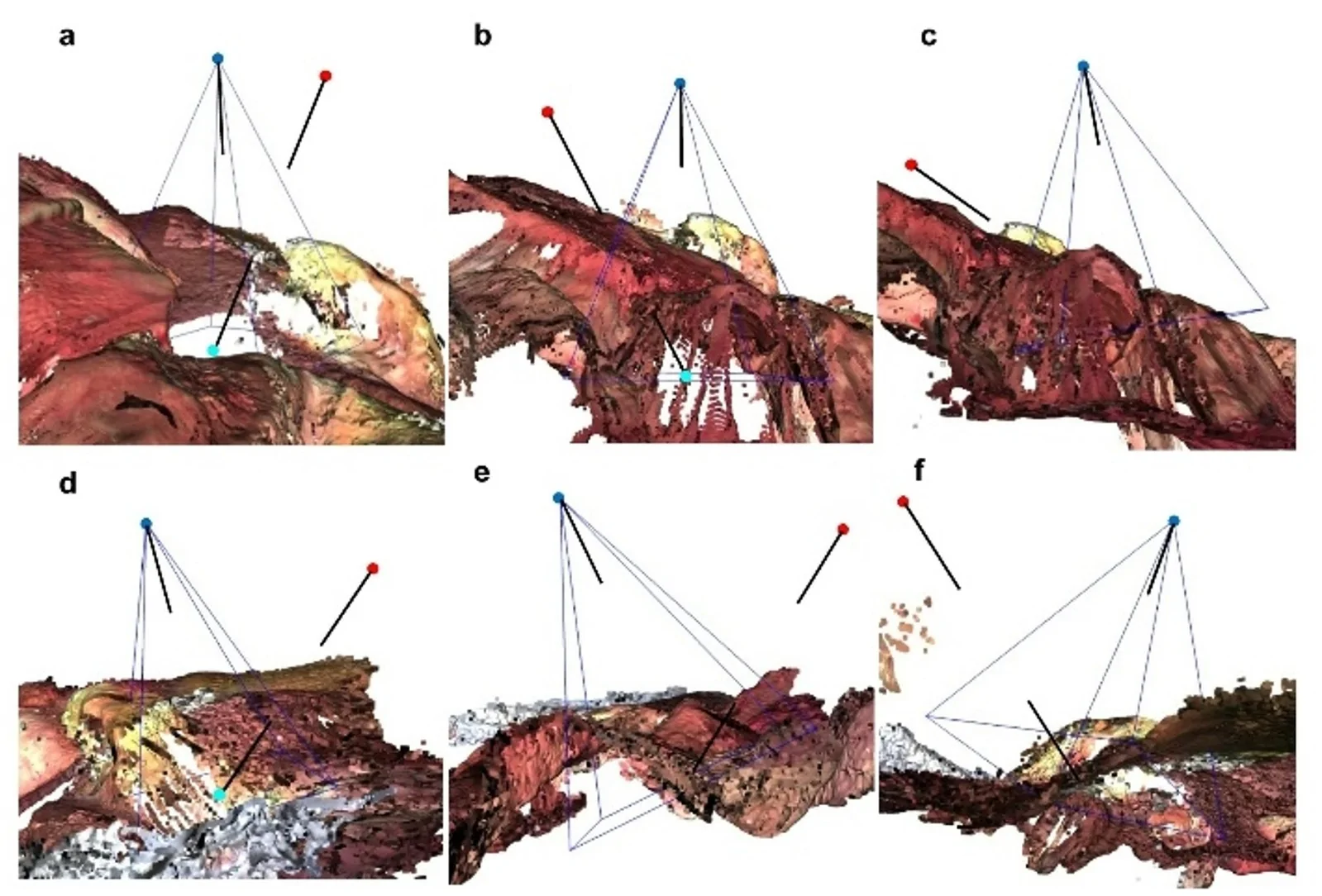
Screenshot of point cloud #5 with a partial side view (different viewing angle compared to the views in Fig. 7) at hole number 1 (**a**), at hole number 2 (**b**), at hole number 3 (**c**), at hole number 4 (**d**), at hole number 5 (**e**), at hole number 6 (**f**). In all six partial views the following information is marked in colors: the detected hole centers (turquoise), corresponding normal vectors (black), ideal laparoscope position (red) and viewing direction (black), and the improved laparoscope viewpoint including FOV pyramid (blue) and viewing direction (black). Due to occlusions, the hole centers and normal vectors are visible only partially in (**c**, **e**, **f**).

Hole number 1 is placed in the middle of the 3D model, and the search algorithm found a solution (Fig. 6a). The camera’s FoV fully covers the area of the hole. The computation time was 0.5 s and the angle between ideal and improved laparoscope viewpoint is small (Fig. 7a). Holes 2, 3, 4, 5, and 6 (Fig. 6b – f) lie in the outer region of the 3D model and were more challenging to reach. The search algorithm found a solution for the four holes and the camera’s FoV successfully captures the ROI. Different to number 1, number 2–6 required a longer computation time and have a greater angle between ideal and improved laparoscope viewpoint (Fig. 7b – f).

Hole number 7 is the only one of point cloud #5 without an improved viewpoint and therefore cannot be repaired. This hole is difficult to reach because it lies below the trocar and its directional vector points horizontally (see Fig. 5).

The next validation step is the quantitative improvement of the hole repair pipeline and therefore, the analysis of the surface coverage and the reconstruction error. In each dataset from #8 to #14, one non-improved point cloud is available, which contains the prepared holes. Then, one improved point cloud is generated by applying the brute-force viewpoint search, and one improved point cloud is generated by applying the ideal laparoscope position – explained in the section Methodology, for which we must avoid the RCM constraint. The latter represents our baseline approach, and all results are summarized in Table 2. The first column states the point cloud number, the next four columns state the surface coverage in % with a threshold of 2 mm for the non-improved, baseline, brute-force based point clouds, and the delta between brute-force based and non-improved. The last four columns list the RMSE of the reconstruction error in mm for the point clouds and delta analogously to the previous four columns. The best results (high coverage and low RMSE) are highlighted in bold.

The surface coverage increased after applying the brute-force based approach in all seven datasets. Four times the brute-force approach outperforms the baseline (dataset #8, #11, #13, #14). In three datasets #9, #10, #12, the baseline method achieved higher coverages. The delta between the brute-force and the non-improved method is between + 0.6% to + 5.0%, which results in a relative improvement of the surface coverage between + 0.8% and 6.3%. The RMSE remains stable over the three different categories and deviates between brute-force and non-improved in case of #10, #12, #13, and #14 around − 0.1 and + 0.4 mm. Overall, the RMSE is low with values between 1.0 and 2.4 mm.

**Table 2:** Summary of the quantitative evaluation results for the additional seven point clouds of ex vivo porcine organs, listing the surface coverage in % and the RMSE of the reconstruction error in mm for the non-improved point cloud (Original), the improved point cloud through the baseline method (Baseline), and the brute-force based search approach (Brute-force), including a column representing the delta between the original and improved point cloud.

Point cloud	Surface coverage in % with $\:{\uptau\:}=2\:\mathrm{m}\mathrm{m}$			Relative improvement of surface coverage of brute-force vs. non-improved in %	RMSE in mm			
	Non-improved	Baseline	Brute-force		Non-improved	Baseline	Brute-force	Delta Brute-force vs. non-improved
#8	90.6	91.5	**91.6**	+ 1.1	1.3	1.3	**1.3**	0.0
#9	90.1	**93.5**	92.2	+ 2.3	1.0	1.1	**1.0**	0.0
#10	79.9	**85.3**	84.9	+ 6.3	**1.3**	1.5	1.7	+ 0.4
#11	76.8	80.2	**80.5**	+ 4.8	1.8	1.9	**1.8**	0.0
#12	72.0	**76.3**	74.9	+ 4.0	**2.2**	2.3	2.4	+ 0.2
#13	78.9	79.4	**79.5**	+ 0.8	1.6	1.5	**1.5**	-0.1
#14	74.3	77.2	**77.3**	+ 4.0	**1.7**	1.8	1.9	+ 0.2

**Fig. 8:**
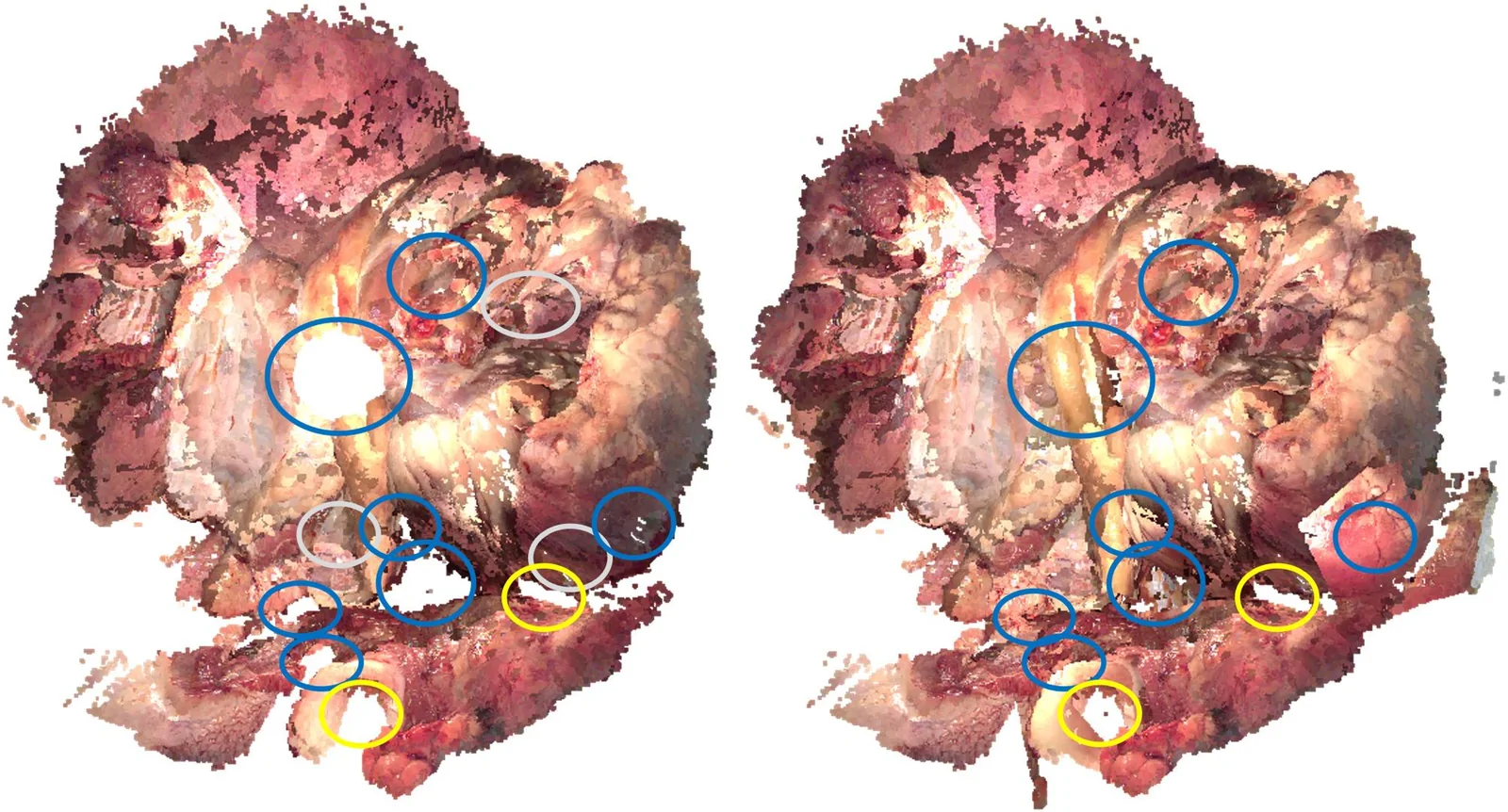
Screenshot of point cloud #10 from a frontal view in the non-improved state (left) and after hole repair (right). It is visible that the hole repair method successfully repaired some of the prepared holes, which are marked by blue circles. The geometrically unreachable holes are marked by grey circles, and the yellow circles show areas, which remain undetected by the hole detection method.

In addition to the quantitative metrics, the qualitative improvement of the point cloud before and after hole repair is evaluated. Figure 8 shows the point cloud of dataset #10 from the front view before (left) and after (right) hole repair based on the brute-force search. In sum, ten holes were automatically detected, which are marked by blue and grey circles, while two holes remained undetected (yellow circles). For three of the ten prepared holes the viewpoint could not be improved, so that a total of seven holes could be successfully repaired by the brute-force approach. The appearance of the point cloud on the right improved and looks more complete compared to the one on the left. Some holes are not visible from the frontal view.

The computation time varies from hole to hole depending on the hole’s position and normal vector relative to the trocar point’s position. Holes placed in the outer circle of the 3D reconstruction – further away from the trocar point – are more difficult to be reached, especially if the normal vector points in the direction away from the trocar point. Holes, which are placed in the inner circle – closer to the trocar point – can be reached more easily, except for those holes directly below the trocar point and with normals being horizontally. The viewpoint search calculation of easier reachable ROIs requires 0.5–2 s, while difficult reachable ROIs require up to 40 s. The average of all holes in the seven evaluated point clouds is ten seconds computation time.

To improve computational performance, we formulated the laparoscope position search as a nonlinear least-squares problem and solved it through the scipy.optimize library in Python with the Trust Region Reflective (trf) algorithm and an initial guess of 0.2 rad for $\:\theta\:$, 0.0 rad for $\:\phi\:$, and 0.0 rad for $\:\psi\:$. The minimization problem is the same as described in the methodology section. We evaluated this on one additional dataset containing seven prepared holes, while the optimizer could successfully find a solution for six of them, the seventh hole could not be optimized. The surface coverage lies at 68.8% for the original point cloud (RMSE at 1.9 mm), at 74.9% for the baseline point cloud (RMSE 2.1 mm), and at 73.6% for the nonlinear optimized point cloud (RMSE at 2.1 mm). The average computation time per hole lies at 6 ms and therefore, a nonlinear optimization solver provides a time-relevant solution.

## Discussion

The approach presented in this work tackles the challenge of autonomous rigid laparoscope navigation under RCM constraint. The scope of it is to guide the laparoscope autonomously, which then visualizes a target ROI defined by a key point, while the key point is selected in a previous 3D reconstruction from laparoscopic images. The search algorithm finds candidate points on a virtual spherical section surface, which is placed around the key point and whose alignment depends on the key point’s directional vector. If successful, an improved laparoscope viewpoint is calculated, and the robot guides the laparoscope to that position. The evaluation of our method was done by applying it on 14 point clouds of ex vivo porcine organs. These point clouds contain prepared holes and provide one key point and one directional vector per hole. The results show that for 85% of prepared holes, an improved laparoscope viewpoint could be found, all of them reachable in terms of inverse kinematics of the UR5 CB3. For the other 15% of the prepared holes, our search approach could not find any solution. The new scanned areas are integrated manually as individual point clouds to repair holes in the 3D reconstruction. Qualitatively, the 3D reconstruction looks more complete after hole repair. Quantitatively, the reconstruction error after hole repair remains low and the surface coverage rises between 1 and 6.3%.

The results fell short of our expectations, which was an increase in coverage of at least 10%.

An analysis of failure cases shows following reasons:

1. Kinematic constraints restrict necessary FOV.
2. Low image quality, e.g. due to light reflections, causes incomplete point clouds.
3. Angle limit of 45° for $\:\theta\:$ not sufficient in all cases.
4. Obstructions hiding the ROI, e.g. due to undercuts.

**Fig. 9:**
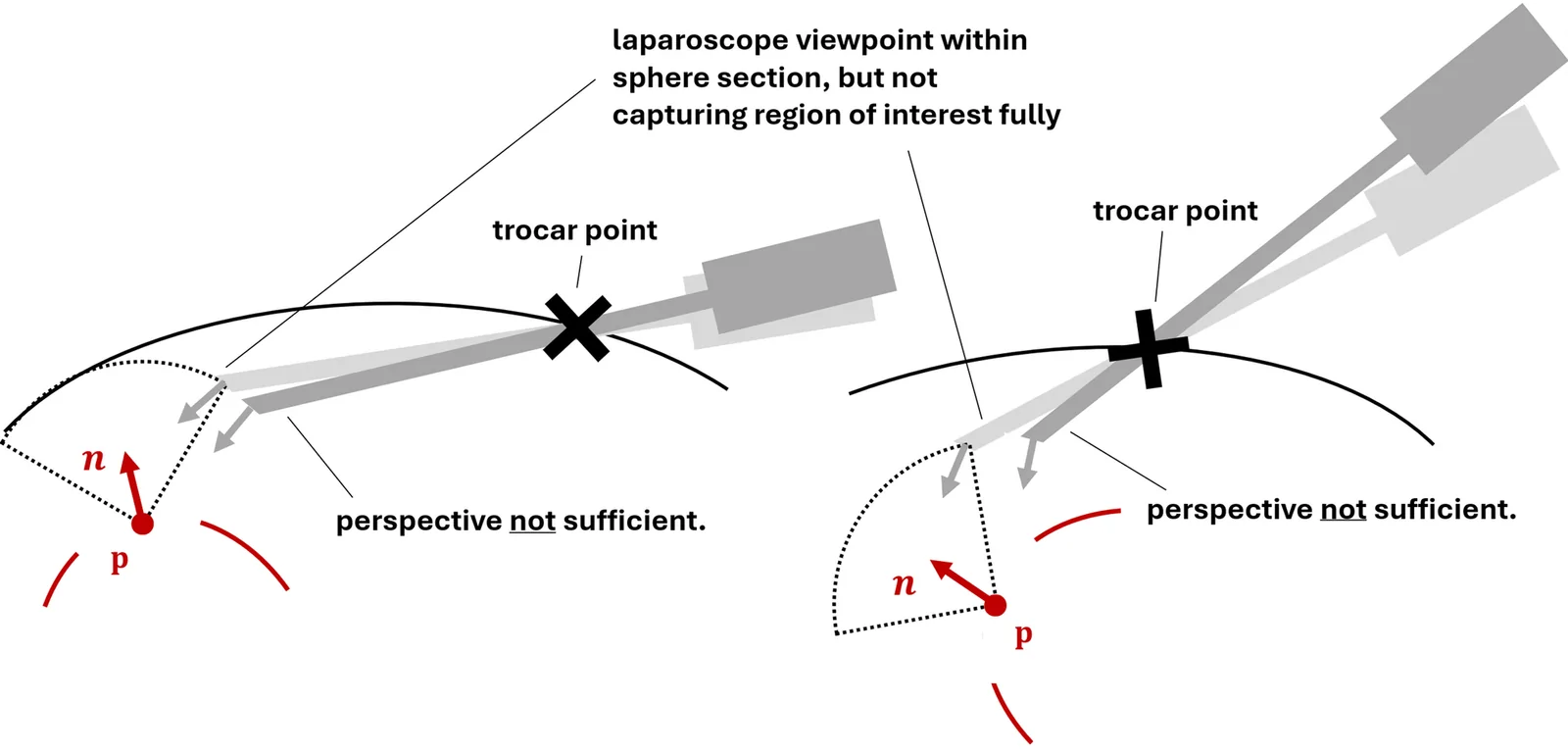
Sketch of spherical section-based search for laparoscope viewpoints for unreachable regions of interest. One unreachable situation is due to a region of interest, represented by the point $\:\mathbf{p}$ and normal vector $\:\boldsymbol{n}$, too far away from the trocar point (left) and the other unreachable situation due to a directional vector $\:\boldsymbol{n}$ pointing away from the trocar point and being too horizontally.

The kinematic constraints lead to scenarios in which the ROI is impossible to reach. An influencing factor for the successful calculation of improved laparoscope viewpoints is the ROI position and the normal vector’s direction relative to the position of the trocar point. A ROI in the outer area of the organ model means a longer distance from the trocar point, which requires a long insertion of the laparoscope into the abdomen. At the same time, it requires the rigid laparoscope to be angled in a certain way to capture the ROI, which is not reachable under trocar point consideration (see Fig. 9 on the left). Other examples of an unreachable ROI is a directional vector pointing away from the trocar as demonstrated in Fig. 9 on the right, or a directional vector pointing horizontally as hole number 7 shown in the section Results in Fig. 5. In such cases, the system limit is reached, and a sufficient viewpoint is impossible. An additional DOF would solve this problem, either through a flexible tip or an additional joint making the rigid tip adjustable within the abdomen.

**Fig. 10:**
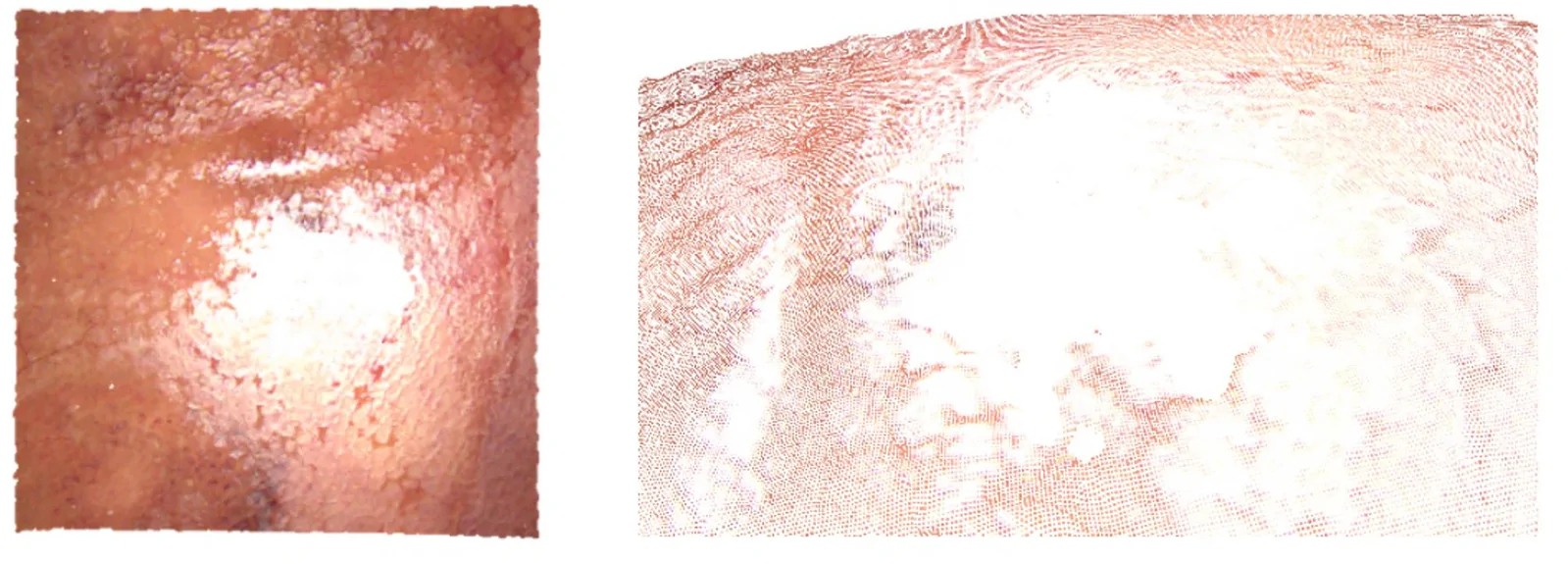
Two screenshots of the individual point cloud to repair hole number 1 in dataset #13 from the baseline approach. The white area in the middle of the point cloud (left) and in the zoomed view (right) represents nan values caused by light reflections.

The image quality has a direct effect on the coverage of each individual point cloud. In the case of reflections or shadows, stereo matching algorithms have difficulties finding corresponding point pairs in the stereo images, which leads to pixels without depth information and following, to incomplete point clouds, as presented in Fig. 10.

**Fig. 11:**
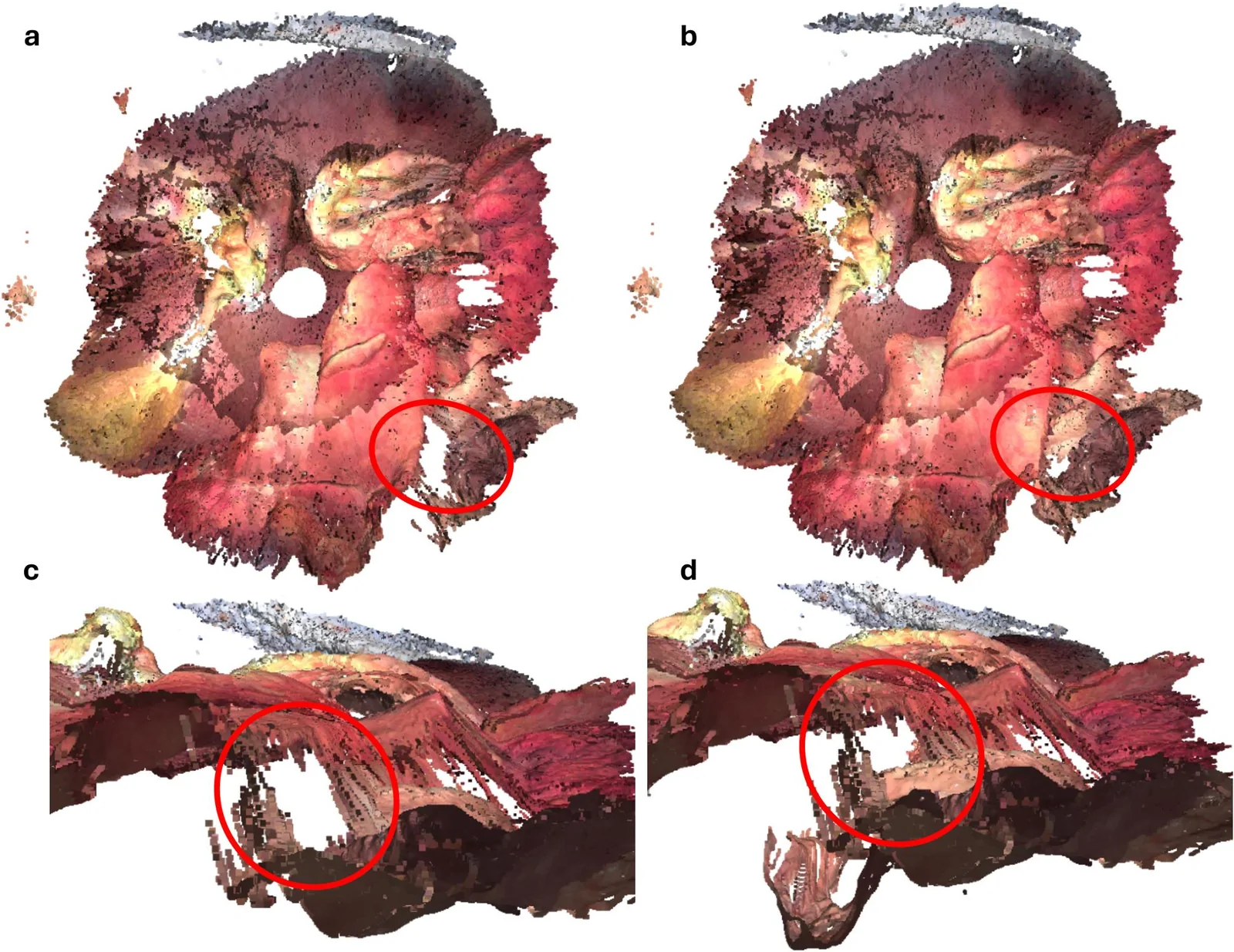
Screenshot of point cloud #5 from the front view (**a** and **b**) and a partial side view (**c** and **d**). Both screenshots on the left (**a** and **c**) show the point cloud without hole repair, while the screenshots on the right show the point cloud with hole repair. The individual point cloud, which was captured after the laparoscope viewpoint search for hole number 5 (see Figs. 7e and 5e) was manually integrated into the previous 3D reconstruction.

The angle limit of 45° for $\:\theta\:$ is not sufficient in all hole scenarios, especially in areas with steep slopes as in Fig. 11, hole number 5 marked by a red circle. While Fig. 11a, and Fig. 11c present the view at the point cloud without hole repair, Fig. 11b, and Fig. 11d show it after hole repair. From the front view (Fig. 11 a, b), hole number 5 seems to be successfully filled, whereas the side view reveals that missing areas remain. Our explanation is that the region of this hole is steep and the camera’s viewpoint does not capture the slope as it would, if the camera was positioned perpendicular to the slope. Following, our approach is not sufficient for some ROI and the angle should rather be restricted to 30° with the result of more failure cases for the laparoscope position search.

**Fig. 12:**
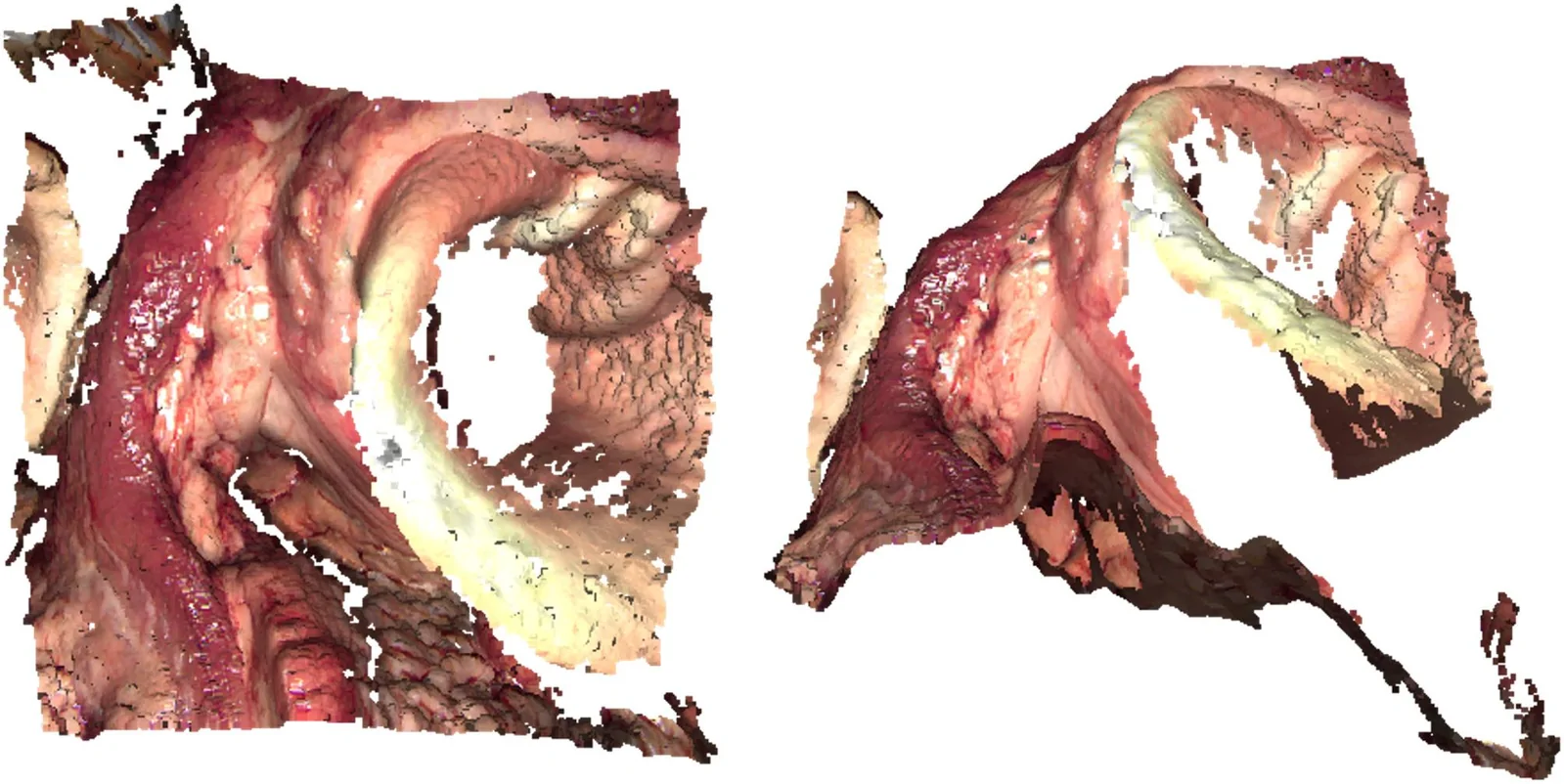
Screenshot of hole number 2 of point cloud #10 from the front view (left) and a side view (right). It shows part of the esophagus.

Some organs have textures that act as obstacles and hide textures below them, which then leads to incomplete point clouds, as in Fig. 12. The end of the esophagus (bright yellow ring) hides the textures below it, which becomes obvious with all the white areas visible in the side view of Fig. 12 on the right. To capture this region, the obstacle should be considered when deciding for a laparoscope position. In this case, a better laparoscope position is not possible without colliding with neighboring organ texture. A mitigation strategy would be a camera system with more degrees of freedom, especially within the abdomen between trocar point and laparoscope tip.

Our 3D reconstruction approach is based on a two-stage procedure: a first rough and a second detailed scan. We claim that this is risk-minimizing and safer. Every intervention is different, as the exact positioning of the trocar point relative to the organs, and the thickness of the patient’s abdominal wall. It would be risky to follow a detailed trajectory in the first scan to reach all necessary viewpoints without having seen the actual organ location, as collisions between laparoscope and organs or between laparoscope and abdominal wall could occur. Therefore, we suggest a coarse scan from laparoscopic viewpoints near the trocar with only small insertion depths, generating a bird-view at the surgery scenery and creating a rough 3D reconstruction. Based on this, trajectories for hole repair and other following steps can be calculated.

Our search strategy showed that autonomous laparoscope guidance based on 3D reconstruction can acquire additional image data and thereby improve surface coverage. Nevertheless, several limitations remain and could not be addressed within this work.

1. Point cloud alignment and integration still require manual intervention.
2. The observed acceleration of nonlinear optimization is preliminary.
3. The trocar position is assumed to be a fixed point.
4. Experimental validation is limited to ex vivo datasets.
5. Complete surface coverage (100%) could not be achieved.

Our current approach is not yet fully autonomous. Although the laparoscope is guided autonomously by the robot and captures new image data, the integration of the new scans into the existing point cloud is still performed manually. Originally, our approach called for initial positioning of new scans based on the robot’s flange position, followed by fine registration using ICP, which would have taken place without any manual intervention. However, this led to an increase in the reconstruction error, which is why we opted for manual initial alignment followed by ICP registration. Alternative global registration methods that could offer autonomous solutions have not yet been tested in this experiment but are planned.

We evaluated the nonlinear optimization solver as an alternative to the brute-force viewpoint search by using a single dataset. The results indicate a promising reduction in computation time from approximately 10 s to 6 ms. But these findings should be considered preliminary, because the current evaluation does not capture variations in anatomical geometry, trocar position or reconstruction conditions that may influence optimization performance. To assess robustness and generalizability of the proposed optimization solver, future studies should evaluate the method across multiple datasets exhibiting the variations described above.

In the experimental set-up of this study, we defined the trocar as a fixed point. In contrast to this, in practical use, the trocar can slightly shift due to deformations of the patient’s abdominal wall, which would have an impact on the laparoscope position search. On the one hand, our approach could benefit from a slight shift of the trocar because it loosens the RCM constraint and provides an increased solution space. On the other hand, an unknown shift of a few millimeters changes the kinematic situation and could lead to an unintended shift in the camera’s FOV, and therefore, to an erroneous hole repair. Following, the results of this study are limited to the premise of a fixed trocar point, and future experiments should consider a tolerance range of the trocar position.

The experimental validation was limited to ex vivo datasets. Nevertheless, the proposed framework addresses a task that is relevant in clinical practice. Since the viewpoint search relies primarily on geometric information extracted from a reconstructed model, the underlying concept is not restricted to ex vivo conditions and could in principle be transferred to in vivo procedures. However, it currently excludes challenges that exist in a real surgical scene, such as tissue motion caused by peristalsis, respiration, and heartbeat, as well as visual disturbances such as blood, smoke, specular reflections, and contamination of the laparoscope lens. Large tissue movements are expected to have the strongest impact on our approach, as they change the geometry of the reconstructed scene and therefore might require a repeated or continuous update of the 3D reconstruction. Periodic breathing-induced motion could be compensated for by phase estimation, while heartbeat-induced motion is expected to be less relevant. Reflections were partially represented in our experimental set-up due to ex vivo organs with a moist surface. The presence of blood, smoke, and contamination can reduce image quality and 3D reconstruction accuracy. In clinical practice, such effects are mitigated by suction, smoke evacuation, or lens cleaning. To avoid such situations altogether, we recommend that the proposed scanning procedure should be executed as the initial step of an intervention directly after introducing the laparoscope into the abdomen and before surgical manipulation is performed.

Complete surface coverage (100%) could not be achieved in this study, and, as previously explained, the increase in coverage was relatively small in some cases due to different failure cases. Of the remaining 10–20% needed to achieve 100% coverage, 1–5% was covered in absolute terms, which corresponds to a relative increase of 1-6.3% and 10–25% of the remaining amount. Furthermore, the proposed method did not consistently outperform the baseline. This is not unexpected, as the baseline assumes an ideal viewpoint directly facing the ROI, whereas our method is restricted by the trocar constraint. As a result, some viewpoints that would provide a better view of missing regions are simply not reachable under this constraint. Nevertheless, the method can still be useful in practice. It identifies regions that are insufficiently represented in the current reconstruction and autonomously guides the laparoscope towards viewpoints that provide additional image and point cloud information. Even though the overall increase in coverage is limited, previously unseen or poorly reconstructed areas can be observed from new angles, leading to a more complete representation of the scene and potentially reducing blind spots.

All selected viewpoints were found to be IK-feasible. Thus, the experimental results do not demonstrate a measurable benefit of the IK constraint in the investigated datasets. This might be because the evaluated target regions were located within the robot’s workspace and could be reached without violating kinematic limits. However, the IK constraint remains an important component of the framework because without feasibility verification, the search algorithm could select viewpoints that cannot be physically reached by the robot. The relevance of this constraint could increase in situations with extreme viewing directions, alternative trocar locations, or robot systems with a more restricted workspace.

## Conclusion

In this work, we presented a novel trocar-constrained laparoscope viewpoint search approach to enhance the surface coverage in 3D reconstruction in robot-assisted laparoscopy formulated as a geometric problem. The method specifically addresses holes in existing 3D reconstruction, and we showed in an experimental validation with ex vivo porcine organs, that our method improves the appearance of the point cloud by acquiring additional scans of previously uncovered regions, and improves the surface coverage by up to 6.3% while keeping the reconstruction error at the same low level (RMSE at around 1–2 mm). Currently the computation time of 10 s on average is too high for practical use, therefore, we tested our approach with a nonlinear optimization solver on one dataset, achieving 6 ms on average. As a next step, the registration logic for integrating new scans must be automated to avoid any manual interaction. The results did not improve more than 6.3% in surface coverage, which only leads to an absolute coverage of 75–92%. In future projects, our approach must be further improved to reach higher coverage, and the nonlinear optimization solver will be tested with more datasets to validate its robustness and generalizability.

## Data Availability

The datasets generated and analyzed during the current study are available from the corresponding author upon request.

## Appendix

The rotation matrix $\:{{}^{\mathrm{w}\mathrm{o}\mathrm{r}\mathrm{l}\mathrm{d}}\mathbf{R}}_{\mathrm{s}\mathrm{p}\mathrm{h}\mathrm{e}\mathrm{r}\mathrm{e}}^{}$ is calculated based on the rotation. vector between the world frame’s z-axis direction $\:{\widehat{\boldsymbol{e}}}_{\boldsymbol{z}}$ and the normal vector $\:\boldsymbol{n}$ (approach after Rodrigues’ rotation formula and known as axis angle representation). First, the angle $\:\theta\:$ between both vectors is determined by the relation between a scalar product and an angle, as in Eq. (10).

10 $$\:\theta\:={\mathrm{cos}}^{-1}\frac{{\widehat{\boldsymbol{e}}}_{{\boldsymbol{z}}_{\boldsymbol{w}\boldsymbol{o}\boldsymbol{r}\boldsymbol{l}\boldsymbol{d}}}\:\boldsymbol{n}}{\left|{\widehat{\boldsymbol{e}}}_{{\boldsymbol{z}}_{\boldsymbol{w}\boldsymbol{o}\boldsymbol{r}\boldsymbol{l}\boldsymbol{d}}}\right|\left|\boldsymbol{n}\right|}$$

Secondly the cross product $\:{\boldsymbol{v}}_{\boldsymbol{c}\boldsymbol{r}\boldsymbol{o}\boldsymbol{s}\boldsymbol{s}}$ of both vectors is calculated, as in Eq. (11).

11 $$\:{\boldsymbol{v}}_{\boldsymbol{c}\boldsymbol{r}\boldsymbol{o}\boldsymbol{s}\boldsymbol{s}}={\widehat{\boldsymbol{e}}}_{{\boldsymbol{z}}_{\boldsymbol{w}\boldsymbol{o}\boldsymbol{r}\boldsymbol{l}\boldsymbol{d}}}\times\:\boldsymbol{n}$$

$\:\theta\:$. and $\:{\boldsymbol{v}}_{\boldsymbol{c}\boldsymbol{r}\boldsymbol{o}\boldsymbol{s}\boldsymbol{s}}$ build the rotation vector $\:{\boldsymbol{v}}_{\boldsymbol{r}\boldsymbol{o}\boldsymbol{t}}$ as in Eq. (12).

12 $$\:{\boldsymbol{v}}_{\boldsymbol{r}\boldsymbol{o}\boldsymbol{t}}=\theta\:\cdot\:\:{\boldsymbol{v}}_{\boldsymbol{c}\boldsymbol{r}\boldsymbol{o}\boldsymbol{s}\boldsymbol{s}}$$

The resulting rotation. vector is transformed into the rotation matrix $\:{{}^{\mathrm{w}\mathrm{o}\mathrm{r}\mathrm{l}\mathrm{d}}\mathbf{R}}_{\mathrm{s}\mathrm{p}\mathrm{h}\mathrm{e}\mathrm{r}\mathrm{e}}^{}$ after Rodrigue’s rotation formula (Eq. 13).

13 $$\:{{}^{\mathrm{w}\mathrm{o}\mathrm{r}\mathrm{l}\mathrm{d}}\mathbf{R}}_{\mathrm{s}\mathrm{p}\mathrm{h}\mathrm{e}\mathrm{r}\mathrm{e}}^{}=\mathrm{c}\mathrm{o}\mathrm{n}\mathrm{v}\mathrm{e}\mathrm{r}\mathrm{t}\_\mathrm{f}\mathrm{r}\mathrm{o}\mathrm{m}\_\mathrm{r}\mathrm{o}\mathrm{t}\mathrm{v}\mathrm{e}\mathrm{c}\_\mathrm{t}\mathrm{o}\_\mathrm{m}\mathrm{a}\mathrm{t}\mathrm{r}\mathrm{i}\mathrm{x}\left({\boldsymbol{v}}_{\boldsymbol{r}\boldsymbol{o}\boldsymbol{t}}\right)$$
